# Data in support of qPCR primer design and verification in a Pink1 −/− rat model of Parkinson disease

**DOI:** 10.1016/j.dib.2016.05.056

**Published:** 2016-05-30

**Authors:** Cynthia A. Kelm-Nelson, Sharon A. Stevenson, Michelle R. Ciucci

**Affiliations:** a Department of Surgery, Division of Otolaryngology, University of Wisconsin-Madison, Madison, WI, USA; b Department of Zoology, University of Wisconsin-Madison, Madison, WI, USA; c Neuroscience Training Program, University of Wisconsin-Madison, Madison, WI, USA; d Department of Communication Sciences and Disorders, University of Wisconsin-Madison, Madison, WI, USA

## Abstract

Datasets provided in this article represent the *Rattus norvegicus* primer design and verification used in *Pink1* −/− and wildtype Long Evans brain tissue. Accessible tables include relevant information, accession numbers, sequences, temperatures and product length, describing primer design specific to the transcript amplification use. Additionally, results of Sanger sequencing of qPCR reaction products (FASTA aligned sequences) are presented for genes of interest. Results and further interpretation and discussion can be found in the original research article “Atp13a2 expression in the periaqueductal gray is decreased in the *Pink1* −/− rat model of Parkinson disease” [1].

Specifications table

**Table:** 

Subject area	*Biology*
More specific subject area	*Neurobiology of disease*
Type of data	*Tables*
How data was acquired	National Center for Biotechnology Information (NCBI) Primer Blast was used to design primers and Sanger sequencing was used for primer confirmation.
Data format	*Raw*
Experimental factors	Netprimer® (PREMIER Biosoft, Palo Alto, CA, USA) was used to examine secondary structure of all primers designed through NCBI Primer Blast to avoid primer products. Non-template controls were run with each primer pair to check for formation of primer-dimers and non-specific amplification products.
Experimental features	Specificity for each primer pair was confirmed using melt curve analysis; all primer runs yielded single peak melt curves indicating amplification of single gene products. Furthermore, the qPCR reaction product for each gene was sequenced using Sanger sequencing with both forward and reverse primers at the University of Wisconsin Biotechnology Center to confirm that sequences match intended targets.
Data source location	*Madison, Wisconsin, USA*
Data accessibility	*Data are within this article*

## Value of the data

• **Data presented here allows for experimental replication.**
• **Data can be used as a benchmark for other researchers using rat brain tissue.**
• **Primers can then be manufactured and used in alternative models of Parkinson disease and then compared to this data set.**

## 1 Data

Table 1 describes the rat (*Rattus norvegicus)* primer information including gene name, gene abbreviation, GenBank® accession numbers, experimental primer sequences, melt temperature and product length (base pairs) for each gene (*Pink1*, *Asyn*, *Th*, *D1*, *D2*, *Atp13a2*, *Gba*, *Cflar*, *Gabrb2*, *Gad1*, *Gad2*) as well as reference genes (*Gapdh*, *βactin*).

Table 2 describes the results of Sanger sequencing of qPCR reaction product for each amplification product from the University of Wisconsin Biotechnology Center. Confirmed results presented are FASTA sequences confirmed through NCBI Nucleotide BLAST software.

## 2 Experimental design, materials and methods

Netprimer (PREMIER Biosoft, Palo Alto, CA, USA) was used to examine secondary structure of all primers designed through NCBI Primer Blast to avoid primer products (Table 1). The Pink1 gene primer was used based on a previous publication [2]. Non-template controls were run with each primer pair to check for formation of primer-dimers and non-specific amplification products. Specificity for each primer pair was confirmed using melt curve analysis; all primer runs yielded single peak melt curves indicating amplification of single gene products. Furthermore, the qPCR reaction product for each gene was sequenced using Sanger sequencing with both forward and reverse primers at the University of Wisconsin Biotechnology Center (Table 2). FASTA sequences were entered into the NCBI Nucleotide BLAST software to confirm that sequences matched intended targets.

## Figures and Tables

**Table 1:** *Rattus norvegicus* primer information.

Gene	Gene abbreviation	Accession number	Direction	Sequences	T (°C)	Product (bp)
Gapdh glyceraldehyde-3-phosphate dehydrogenase	*Gapdh*	NM_017008.4	Forward	GGATACTGAGAGCAAGAGAGA	59	106
			Reverse	TTATGGGGTCTGGGATGGAA		
						
Actb actin, beta	*βactin*	NM_031144.3	Forward	TGTGGATTGGTGGCTCTATC	59	149
			Reverse	AGAAAGGGTGTAAAACGCAG		
						
Pink1 PTEN induced putative kinase 1	*Pink1*	Primers created from Dave et al. [2]	Forward	CATGGCTTTGGATGGAGAGT	58	n/a
			Reverse	TGGGAGTTTGCTCTTCAAGG		
						
Snca synuclein, alpha (non-A4 component of amyloid precursor)	*Asyn*	NM_019169.2	Forward	TCAGCCCAGAGCCTTTCAC	58	165
			Reverse	AGCCACAACTCCCTCCTTG		
						
Th tyrosine hydroxylase	*Th*	NM_012740.3	Forward	CTTTGACCCAGACACAGCA	59	123
			Reverse	TGGATACGAGAGGCATAGTTC		
						
Drd1 dopamine receptor D1	*D1*	NM_012546.2	Forward	GCTGGCTCCCTTTCTTCATC	60	111
			Reverse	CACCCAAACCACACAAACAC		
						
Drd2 dopamine receptor D2	*D2*	NM_012547.1	Forward	TCCTTGACCTTCCTCTTGGG	60	188
			Reverse	CCTGACACTGATGTTGCCTG		
						
Atp13a2 ATPase type 13A2	*Atp13a2*	NM_001173432.1	Forward	CTTCTCTCTGTCTGGCTTCC	60	95
			Reverse	TCCTCAGTCCGTTGGTGTAG		
						
Gba glucosidase, beta, acid	*Gba*	NM_001127639.1	Forward	GAGCAGAGTGTTCGGTTAGG	60	115
			Reverse	GATTCAGGGCAAGGTTCCAG		
						
Cflar CASP8 and FADD-like apoptosis regulator	*Cflar*	NM_001033864.2	Forward	GTGCTGCTGATGGAGATTGG	60	107
			Reverse	CTCTTGTCCTTGGCTACCTTG		
						
Gabrb2 gamma-aminobutyric acid (GABA) A receptor, beta 2	*Gabrb2*	NM_012957.2	Forward	GGTGCTTTGTCTTTGTCTTTATGG	61	130
			Reverse	CGCATCTTCTCGTTGTTGG		
						
Gad1 glutamate decarboxylase 1	*Gad1*	NM_017007.1	Forward	GACACTTGAACAGTAGAGACCC	61	116
			Reverse	TGTAGGACGCAGGTTGGTAG		
						
Gad2 glutamate decarboxylase 2	*Gad2*	NM_012563.1	Forward	CCAGGCTCATCGCATTCAC	61	190
			Reverse	GCACTCACCAGGAAAGGAAC		

**Table 2:** Results of Sanger sequencing of qPCR reaction product.

Gene	FASTA (Aligned Sequence)
*Gapdh*	ATCCCAACTCGGCCCCCAACACTGAGCATCTCCCTCACAATTTCCATCCCAGACCCCCATAA
*βactin*	AGATGTGGATCAGCAAGCAGGAGTACGATGAGTCCGGCCCCTCCATCGTGCACCGCAAATGCTTCTAGGCGGACTGTTAC
*PINK1*	CTCTTCTCATTTTTCCCGACCAC
*Asyn*	GGGGAAAACAGGAGGAATCAGAGTTCTGCGGAAGCCTAGAGAGCCGTGTGGAGCAAAGATACATCTTTAGCCATGGATGT
*Th*	CCAGCCTGTGTACTTTGTGTCCGAGAGCTTCAATGACGCCAAGGACAAGCTCAGG
*D1*	GGCTCCCTTTCTTCATCTCGAACTGTATGGTGCCCTTCTGTGGCTCTGAGGAGACCCAGCCAT
*D2*	TTCCTTGACCTTCCTCTTGGGCACAGAAACTAGCTCAGTGGTCGAGCACACCCTGATCGCTGG
*Atp13a2*	CGGTGTCTAAGGGGGCACCCTTCCGCCAGCCGCTCTACACCAACGGACTGAGGAA
*Gba*	GCAACTGTTACCACGTCAATTCCATG
*Cflar*	CTGATGGAGATTGGGGAGAATTTGAATCAATCTGATGTATCCTCCTTAATTT
*Gabrb2*	TCTTCTTTGGGAGAGGACCCCAGCGCCAAAAGAAAGCAGCTGAGAAAGCTGCTAATGCCAACAACGAGAAGATGCG
*Gad1*	GCATCTTCCACGCCTTCGCCTGCAACCTCCTCGAACGCGGGAGCGGATCCTAATACTACCAACCTGCGTCCTACAA
*Gad2*	GCCTTGGGGATCGGAACAGACAGCGTGATTCTGATTAAATGTGATGAGAGAGGGAAAATGATCCCATCTGACCTTGAAAG
