# Supplementary material for: Data in support of qPCR primer design and verification in a Pink1 −/− rat model of Parkinson disease
Source: Data Brief. 2016 May 30;8:360–3. doi: 10.1016/j.dib.2016.05.056 (PMC4909782; doi:10.1016/j.dib.2016.05.056)
Supplement: Supplementary file 1 — Supplementary material [file mmc1.docx]

We have no conflict of interest to report.
